# Phloroglucinol induces apoptosis through the regulation of insulin-like growth factor 1 receptor signaling pathways in human colon cancer HT-29 cells

**DOI:** 10.3892/ijo.2014.2521

**Published:** 2014-06-24

**Authors:** MI-HYE KANG, IN-HYE KIM, TAEK-JEONG NAM

**Affiliations:** Departments of Food and Life Science, Pukyong National University, Nam-gu, Busan 608-737, Republic of Korea

**Keywords:** phloroglucinol, insulin-like growth factor-1 receptor signaling pathway, PI3K/Akt/mTOR signaling pathway, Ras/ERK-MAPK signaling pathway, cell cycle

## Abstract

Phloroglucinol is a polyphenol compound with free radical scavenging, anti-inflammatory and antitumor activity. In this study, we investigated the anticancer effects of phloroglucinol on insulin-like growth factor-1 receptor (IGF-1R) signaling in HT-29 human colon cancer cells. Apoptosis was evaluated using 4′,6-diamidino-2-phenylindole (DAPI) staining, which clearly demonstrated cell shrinkage and condensed nuclei. Treatment with a pan-caspase inhibitor reduced the expression of phosphatidylinositol-3-kinase (PI3K)/Akt, which could induce apoptosis through IGF-1R signaling pathways. Treatment with phloroglucinol significantly inhibited the expression of Ras, Raf, mitogen-activated protein kinase (MEK), extracellular-signal regulated kinase (ERK) phosphorylation, PI3K and Akt. Phloroglucinol also decreased mammalian target of rapamycin (mTOR) and expression of its downstream effectors p70S6 kinase and translation initiation factors elF4B and RPS6. These results demonstrate that IGF-1R activates PI3K/Akt/mTOR and Ras/ERK-MAPK downstream signaling pathways, which has important implications for understanding the roles of cell growth pathways in colon cancer cell tumorigenesis.

## Introduction

Although several therapeutically useful compounds have been identified, their pharmacological properties have been reported using crude extracts (1,2). Phloroglucinol is a multipurpose compound with diverse application as an anti-inflammatory with anticarcinogenic properties that has attracted recent biomedical research interest (3,4). The etiology of cancer involves the suppression of apoptotic processes and dysregulation of cellular proliferation, leading to tumor development (5). The majority of cancer treatment approaches, such as chemotherapy and radiation therapy, destroy cancer cells by inducing apoptosis (6,7). However, cancer cells often develop resistance and many therapies indirectly activate apoptosis by damaging DNA (8,9).

Insulin-like growth factor-1 (IGF-1) signaling promotes cell growth and is used as a crucial proliferative marker in cancer cells (10,11). The IGF-1 receptor (IGF-1R) has a dominant role in cancer cell survival via autocrine and paracrine mechanisms to promote oncogenesis (12,13). Recruitment of these molecules activates phosphatidylinositol-3-kinase (PI3K)/Akt and Ras/extracellular signal-regulated kinase (ERK)-mitogen-activated protein kinase (MAPK) signaling pathways (14).

Activation of PI3K converts phosphatidylinositol 4,5-biphosphate (PIP2) into phosphatidylinositol 3,4,5 phosphate (PIP3), which leads to plasma membrane localization of phosphoinositol-dependent kinase-1 (PDK1) via its pleckstrin homology (PH) domain (15). Akt is the major mediator of PI3K signaling with a large number of downstream substrates, including mammalian target of rapamycin (mTOR) (16).

Furthermore, cross-talk between the PI3K/Akt and the Ras/ERK-MAPK pathways is involved in protein translation (17). mTOR and p70S6 kinase (p70S6K) are the primary downstream effectors that activate translation initiation factors and inactivate regulatory proteins targeting translation (18). During apoptosis, Cyclin D activates the cyclin-dependent kinases (Cdks), such as Cdk4 and Cdk6, which mediates oncogenic actions and provides an attractive therapeutic target (19). The current study aimed to investigate the role of phloroglucinol on apoptosis and IGF-1R signaling pathways in HT-29 colon cancer cells as a potential therapeutic target in cancer therapy.

## Materials and methods

### Cell culture

HT-29 colon cancer cells (ATCC HTB-38; ATCC, Manassas, VA, USA) were grown in 100-mm culture dishes, containing RPMI-1640 medium supplemented with 10% fetal bovine serum (FBS) (HyClone, Logan, UT, USA) and penicillin/streptomycin (P/S). The cells were maintained in a humidified environment under a 5% CO_2_, 95% air at 37°C. The medium was replaced every 2 days and trypsinized when the cells reached 80% confluence.

### Western blot analysis

HT-29 cells were cultured to 60% confluence and then incubated in serum-free medium (SFM) for 6 h. Phloroglucinol (0–50 μg/ml) was added to SFM for an additional 24 h. Cell extracts were prepared by washing cultures with phosphate-buffered saline (PBS) and suspending in extraction lysis buffer [20 mM Tris-HCl (pH 7.4), 150 mM NaCl, 1% NP-40, 1 mM EGTA, 1 mM EDTA, 0.25% Na-deoxycholate, 2.5 mM sodium pyrophosphate] containing protease inhibitors (1 mM sodium orthovanadate, 1 μg/ml aprotinin, 1 μg/ml pepstatin, 1 μg/ml leupeptin, 1 mM NaF, 1 mM PMSF) on ice. Cell extracts were centrifuged at 12,000 rpm for 10 min and the supernatant was used for western blotting. Sample buffer was added to the total cell lysate, and the mixed samples were boiled for 10 min at 100°C. Proteins were separated in 5–15% SDS-PAGE and transferred onto polyvinylidene fluoride membranes (Millipore, Billerica, MA, USA). Membranes were blocked for 90 min at room temperature in blocking buffer [1% bovine serum albumin (BSA) in TBS-T], followed by incubation with primary antibodies (1:1,000 in 1% BSA/TBS-T) overnight at 4°C or for 150 min at room temperature. The membranes were then washed three times for 10 min in TBS-T and incubated with horseradish peroxidase-conjugated goat, mouse, or rabbit secondary antibodies (1:10,000 in 1% BSA/TBS-T). Immunoreactive bands were detected using an enhanced chemiluminescence western blotting kit (Amersham Biosciences, Piscataway, NJ, USA).

### Cell cycle analysis

The effect of phloroglucinol on cell cycle progression was determined using a Muse™ cell cycle analyzer from Millipore (EMD Millipore Co., Hayward, CA, USA). The cells were cultured in 6-well plates grown to 60% confluence and incubated in SFM for 6 h, followed by incubation with phloroglucinol (0–50 μg/ml) for 24 h. Cells were collected in 1% FBS-RPMI-1640 medium, and test reagent was added to each respective tube. Cells were mixed by vortexing and the reaction was incubated for 30 min at room temperature in the dark. Following treatment, cells were stained to evaluate the G0/G1, S and G2/M phase rates.

### mRNA expression analysis

The mRNA expression levels of specific genes were measured by reverse transcription-polymerase chain reaction (RT-PCR). HT-29 cells were seeded onto 6-well plates at 2×10^4^ cells/well in 2 ml of medium. Cells were cultured for 2 days and the medium was replaced with SFM for 6 h, followed by treatment with phloroglucinol (0–50 μg/ml) for 24 h. Cells were treated with 1 ml TRIzol reagent (Invitrogen, Carlsbad, CA, USA), and cDNA was synthesized using the oligo(dt) primer (iNtRON Biotechnology, Seongnam, Korea). The converted cDNA was added to 2X TOPsimple™ DyeMIX-nTaq (Enzynomics, Daejeon, Korea) and the primer (Table I) was dissolved in 0.1% diethylpyrocarbonate (DEPC) water before adding to the reaction mixture. The amplified products were separated on a 1% agarose gel stained with Redsafe™ nucleic acid staining solution (iNtRON Biotechnology).

### 4′,6-Diamidino-2-phenylindole (DAPI) staining assay

The cells were grown to subconfluency on 12-mm coverslips and exposed to phloroglucinol for 24 h. The monolayer of cells was washed with PBS and fixed with 1% paraformaldehyde for 10 min at room temperature. Fixed cells were permeabilized with 0.2% Triton X-100 in PBS for 10 min at room temperature and incubated with 1 μg/ml of DAPI for 5 min. The condensed chromatin and fragmented nuclei in DAPI-stained apoptotic cells were viewed under a confocal microscope.

### Statistical analysis

Study results were analyzed using SPSS software (ver. 18.0; SPSS, Inc., Chicago, IL, USA). Data are presented as means ± standard deviation. Duncan’s multiple range test was used to calculate differences between values. Statistical significance was indicated at P<0.05.

## Results

### Phloroglucinol induces nuclear hallmarks of apoptosis in HT-29 cells

**Figure 1:**
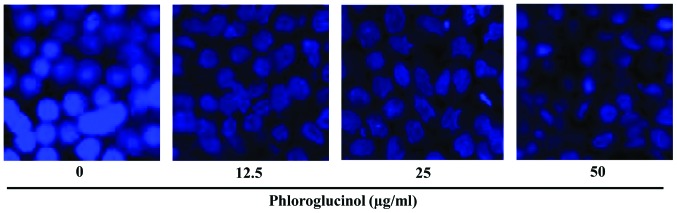
Phloroglucinol-induced apoptosis in HT-29 colon cancer cells. HT-29 cells were treated with phloroglucinol (0, 12.5, 25 and 50 μg/ml) for 24 h before chromatin staining with 4′,6-diamidino-2-phenylindole (DAPI). Apoptotic cells were imaged with a confocal laser microscope using a ×40 objective.

To view apoptotic body formation and nuclear morphological changes characteristic of apoptosis, HT-29 cells were treated with phloroglucinol (0, 12.5, 25 and 50 μg/ml) for 24 h and stained with DAPI. Chromatin was visualized by confocal microscopy. Untreated cells exhibited swelling and increased homogenous chromatin morphology, while phloroglucinol-treated cells exhibited fragmented nuclei with densely stained granular condensed chromatin (Fig. 1).

### Phloroglucinol inhibits HT-29 cell growth by suppressing IGF-1R signaling

**Figure 2:**
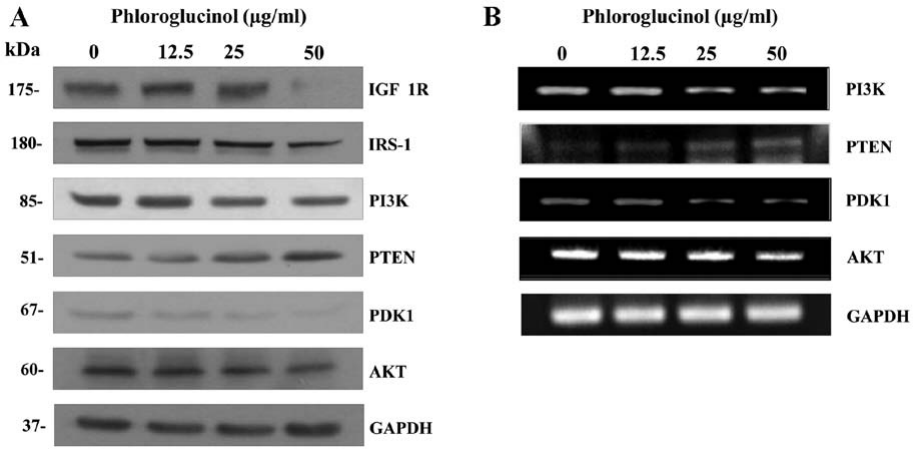
Effect of phloroglucinol on the (A) protein and (B) mRNA expression of IGF-1R, IRS-1, PI3K, PTEN, PDK-1 and Akt in HT-29 cells. Cells were treated with phloroglucinol (0, 12.5, 25 and 50 μg/ml) for 24 h. Glyceraldehyde 3-phosphate dehydrogenase (GAPDH) was used as a loading control. Data are representative of three different experiments.

Activation of IGF-1R signaling includes PI3K and MAPK pathways (20), which can be regulated by IGF binding proteins. To examine the potential role of PI3K and Akt signaling in mediating the effects of phloroglucinol, we performed western blots (Fig. 2A) and RT-PCR (Fig. 2B) analysis. A 24-h treatment with phloroglucinol dose-dependently suppressed expression of IGF-1R protein and downstream signaling proteins such as insulin receptor substrate-1 (IRS-1), PI3K, Akt and PDK1. These results indicate that phloroglucinol could inhibit IGF-1R signaling related molecules, as well as activate the PI3K and Akt pathway.

### Caspase activation inhibits effects of phloroglucinol on IGF-1R-regulated proteins

**Figure 3:**
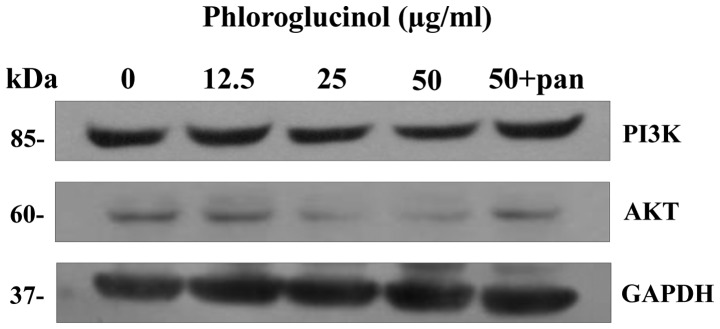
Effect of phloroglucinol on the pan-caspase inhibitor-induced activation of PI3K and Akt in HT-29 cells. Phloroglucinol was added at 0, 12.5, 25 and 50 μg/ml. HT-29 cells treated with 50 μg/ml phloroglucinol were also treated for 1 h with the pan-caspase inhibitor Z-VAD (Ome)-FMK. Each band depicts the quantitative analysis from western blots.

Previous studies have shown that phloroglucinol induced apoptosis via Fas-induced signaling pathways, and results from this study confirmed that phloroglucinol downregulated IGF-1R-related proteins such as PI3K and Akt. To determine whether Fas-mediated apoptosis inhibited IGF-1R signaling pathways, HT-29 cells were treated with a pan-caspase inhibitor, which diminished caspase activation and suppressed PI3K and Akt expression levels (Fig. 3). Thus, IGF-1R signaling is a target of phloroglucinol-induced apoptosis.

### Phloroglucinol inhibits HT-29 cell survival by suppressing mTOR signaling pathways

**Figure 4:**
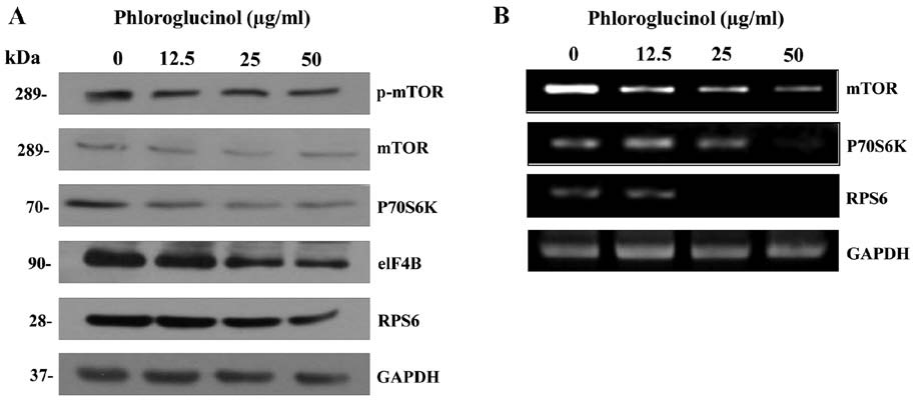
Dose-dependent effect of phloroglucinol on the (A) protein and (B) mRNA expression of mTOR, p70S6K, RPS6 and elF4B in HT-29 colon cancer cells.

Phloroglucinol decreased Akt levels, which has been reported to be phosphatase and tensin homolog (PTEN)-dependent. Therefore, we examined the effect of phloroglucinol on mTOR/p70S6K signaling pathways that regulate cell growth. Phloroglucinol suppressed protein (Fig. 4A) and mRNA (Fig. 4B) expression of mTOR and p70S6K, as well as the translation initiation factors elF4B and RPS6. Collectively, these data indicate that phloroglucinol effectively decreases mTOR/p70S6K growth signaling pathways in colon cancer HT-29 cells.

### Phloroglucinol inhibits HT-29 cell growth by suppressing ERK-MAPK signaling pathways

**Figure 5:**
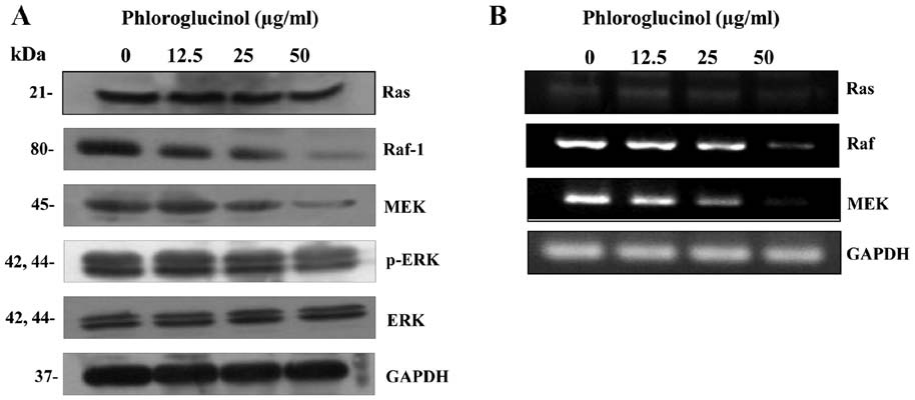
Dose-dependent effect of phloroglucinol on the (A) protein and (B) mRNA expression of Ras, Raf, MEK and phospho-ERK in HT-29 colon cancer cells.

IGF-1R protein and mRNA expression decreased significantly after 24 h of phloroglucinol treatment in HT-29 colon cancer cells. IGF-1R signaling involves activation of Ras, Raf, MEK and ERK (21), and we determined that protein (Fig. 5A) and mRNA (Fig. 5B) expression levels of Ras, Raf, MEK and phosphorylated ERK were suppressed in phloroglucinol-treated HT-29 compared with control cells. These results demonstrate that phloroglucinol inhibits Ras/ERK-MAPK growth signaling pathways in HT-29 cells.

### Effects of phloroglucinol on cell cycle progression in HT-29 cells

**Figure 6:**
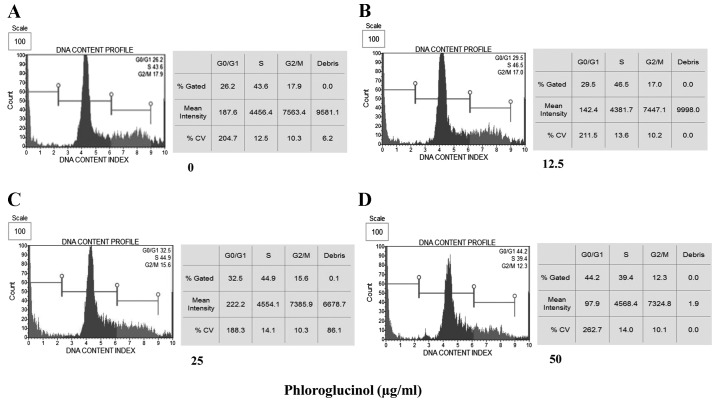
Cell cycle analysis revealed that phloroglucinol induced G0 and G1 cell cycle arrest in a dose-dependent manner in HT-29 cells.

Phloroglucinol-induced apoptosis was sustained via cell cycle arrest (Fig. 6). Phloroglucinol increased the ratio of cells in the G0 and G1 phase, while decreasing the ratio of cells in the G2 and M phases, suggesting that phloroglucinol inhibits cell cycle progression to stimulate apoptosis.

### Effects of phloroglucinol on cell cycle-related proteins

**Figure 7:**
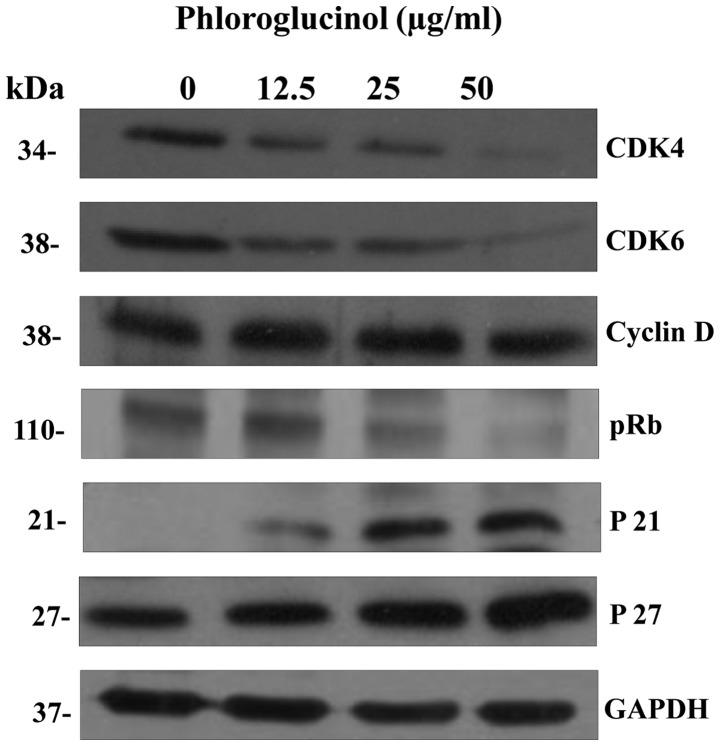
Effect of phloroglucinol on the expression of cell cycle regulated proteins in HT-29 cells. Cells were treated with phloroglucinol (0, 12.5, 25 and 50 μg/ml) for 24 h and total protein extracts were subjected to western blot analysis using antibodies against Cdk4, Cdk6, Cyclin D, pRb, p21 and p27.

To identify the mechanisms mediating phloroglucinol-induced cell cycle arrest, we examined the expression of cell cycle regulatory proteins. Western blot analysis results indicated that the expression of Cdk4, Cdk6, pRb and Cyclin D, which regulate the G0 and G1 phases, decreased with phloroglucinol treatment. However, the levels of p21 and p27 were dose-dependently increased following treatment with phloroglucinol (Fig. 7). Taken collectively, these results demonstrate that phloroglucinol inhibits HT-29 cell proliferation by modulating cell cycle-related proteins.

## Discussion

Previous results suggested that phloroglucinol inhibited growth of HT-29 cells via IGF-1R-mediated signaling pathways. In this study, we confirmed that phloroglucinol induced apoptosis and inhibited cell cycle progression by regulating PI3K/Akt/mTOR and Ras/ERK-MAPK signaling pathways. IGF-1R and apoptotic pathways play important roles in cancer progression (19). Reduced expression of IGF-1R and downstream regulatory proteins inhibits the development of cancer, and the results presented here demonstrated that phloroglucinol inhibited cancer by regulating IGF-1R pathways (Fig. 2). As shown in Fig. 3, a pan-caspase inhibitor suppressed caspase activation and affected the level of IGF-1R-mediated PI3K/Akt signaling proteins. Furthermore, we demonstrated that apoptosis-mediated Fas signaling activity was inhibited by IGF-1R.

IGF and IGF-1 receptors represent a group of biological growth transducers involved in diverse pathological processes (20). IGF-1R is auto-phosphorylated by its intrinsic tyrosine kinase activity to facilitate activation of downstream signaling molecules. Activated lGF-1R and phosphorylated adaptor proteins, such as IRS-1, are coupled to the PI3K/Akt pathway (21,22). PI3K/Akt signaling, along with mTOR/p70S6K, are the primary downstream effectors that activate translation initiation factors and inactivate their regulators (23). In addition, these pathways participate in cross-talk with Ras/ERK-MAPK pathways (24). The present results indicated that phloroglucinol inhibited IGF-1R signaling via mTOR, p70S6K, as well as Ras, Raf, MEK and phosphorylated ERK molecules (Figs. 4 and 5). The study findings also revealed an increase in the percentage of cells in the G0 and G1 phase from 26.2 to 44.2%, while the percentage of cells in the remaining phases decreased following treatment with phloroglucinol from 17.9 to 12.3%, suggesting that phloroglucinol inhibited cell cycle progression (Fig. 6). Phloroglucinol-mediated cell cycle arrest was the result of apoptosis and related to the expression of cyclins and Cdk cell cycle regulatory proteins (Fig. 7). Thus, phloroglucinol-induced cell cycle arrest resulted in cell death in proliferative HT-29 colon cancer cells.

**Figure 8:**
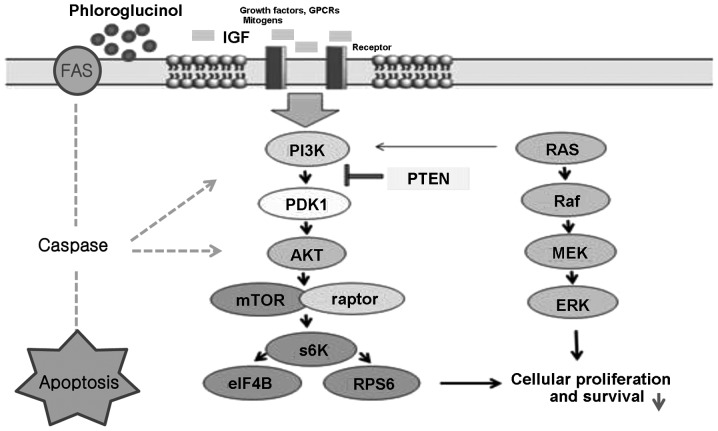
Proposed mechanism of IGF-1R-mediated activation of PI3K/Akt and Ras/ERK-MAPK signaling. The downstream effectors of PI3K/Akt included mTOR/p70S6K and the translation initiation factors elF4B and RPS6.

The extracellular domain of the IGF receptor is responsible for ligand binding, inducing the formation of receptor dimers, and the phosphorylation of tyrosine residues in the cytoplasmic domain of the receptor through activation of an intrinsic kinase. The phosphorylated tyrosine residues play a critical role in intracellular signaling. In cancer cells, IGF-1R activates IRS-1 by stimulating several intracellular signaling proteins such as PI3K and Akt kinase (25–27). Our results provide mechanistic evidence regarding IGF-1R regulated signaling that includes the PI3K/Akt/mTOR and Ras/ERK-MAPK pathways. mTOR is a serine/threonine protein kinase that integrates nutrient availability with growth factor signaling used to modulate cell proliferation and function (28). Interpretation of these results is complicated by the common regulatory molecules involved in apoptosis, such as caspase and the PI3K/Akt/mTOR signaling pathway. The PI3K/Akt/mTOR signaling pathway is known to play a key role in cell survival, differentiation and metabolism. Videlicet, an inhibitor of the PI3K/Akt/mTOR signaling pathway, caused cell death associated with apoptosis (29,30). The current results indicate that IGF-1 receptor regulated Akt and mTOR, as well as other important molecules such as PTEN, PDK-1 and p70S6K involved in PI3K/Akt/mTOR signaling (Fig. 8).

Likewise, Ras signaling is enhanced due to upstream events, such as the activation of IGF-1R (31). Regardless of the mechanism, augmented Ras signaling contributes to a molecular signature for colon cancers. Ras is a significant oncogene product involved in the pathogenesis of cancers, and expression of activated Ras is sufficient to induce oncogenesis in both normal and cancer cells, supporting the use of common downstream pathways (32,33). Ras transmits a signal to the serine/threonine kinase Raf, which subsequently activates MAPK, resulting in cell growth via the transcriptional activation of multiplex targets (34). Our results demonstrated that IGR-1R modulated Ras, Raf, MEK and phosphorylated ERK to suppress the survival of HT-29 colon cancer cells (Fig. 8).

In conclusion, we have demonstrated that phloroglucinol induced apoptosis via an apoptotic pathway involving growth factors, accounting for the effect of phloroglucinol on IGF-1R regulated signaling pathways in HT-29 colon cancer cells. These findings suggest a vital role for IGF-1R in colon cancer tumorigenesis, as well as the potential value of phloroglucinol as a therapeutic agent with anticancer effects on human colon cancer.

## Figures and Tables

**Table I:** Oligonucleotide sequences of the primer used in RT-PCR analyses.

Gene name	Primer sequence
PI3K	
F	5′-AGG AGC GGT ACA GCA AAG AA-3′
R	5′-GCC GAA CAC CTT TTT GAG TC-3′
Akt	
F	5′-CAA CTT CTC TGT GGC GCA GTG-3′
R	5′-GAC AGG TGG AAG AAC AGC TCG-3′
PTEN	
F	5′-TGG AAA GGG ACG AAC TGG TG-3′
R	5′-CAT AGC GCC TCT GAC TGG GA-3′
PDK1	
F	5′-AAG GGT ACG GGC CTC TCA AA-3′
R	5′-CCC ACG TGA TGG ACT CAA AGA-3′
mTOR	
F	5′-CGC TGT CAT CCC TTT ATC G-3′
R	5′-ATG CTC AAA CAC CTC CAC C-3′
p70S6K	
F	5′-TAC TTC GGG TAC TTG GTA A-3′
R	5′-GAT GAA GGG ATG CTT TAC T-3′
RPS6	
F	5′-AAG GAG AGA AGG ATA TTC CTG GAC-3′
R	5′-AGA GAG ATT GAA AAG TTT GCG GAT-3′
Ras	
F	5′-CCC GTC CTC ATG TAC TGG TC-3′
R	5′-ATC TTG GAT ACG GCA GGT CA-3′
Raf	
F	5′-GAT GAT GGC AAA CTC ACG GAT TC-3′
R	5′-AAG GCA GTC GTG CAA GCT CA-3′
MEK	
F	5′-CGATGGATCCCCCAAGAAGAAGCCGAC G-3′
R	5′-CGATCTCGAGTTAGACGCCAGCAGCAT G-3′
GAPDH	
F	5′-CAG CCG AGC CAC ATC G-3′
R	5′-TGA GGC TGT TGT CAT ACT TCT C-3′

F, forward; R, reverse.
